# Exploring the factors affecting preschool educators’ health teaching capacity of life skills using the PRECEDE model: a study of preschool educators in northern Taiwan

**DOI:** 10.1186/s12889-022-13005-2

**Published:** 2022-03-25

**Authors:** Hui Ling Chen, Wei Hsiang Huang, Chieh Hsing Liu

**Affiliations:** 1 Department of Health Promotion and Health Education, College of Education, National Taiwan Normal University, Taipei, Taiwan; 2 Department of Early Childhood Care and Education, University of Kang-Ning, Taipei, Taiwan; 3 Fire Department, New Taipei City Government, New Taipei, Taiwan

**Keywords:** PRECEDE model, Life skills, Health teaching, Preschool educators

## Abstract

**Background:**

The purpose of this study was to apply the Predisposing, Reinforcing, and Enabling Constructs in Educational Diagnosis and Evaluation (PRECEDE) model to analyze the factors influencing preschool educators’ ability to teach health education through life skills.

**Methods:**

This cross-sectional study utilized stratified random sampling and administered survey questionnaires to 503 preschool educators in public and private kindergartens in Taipei City and New Taipei City in 2019. Descriptive and hierarchical regression analyses were conducted. The PRECEDE model demonstrated a significant correlation between the enabling, reinforcing, and predisposing factors explored in this study and the preschool educators’ ability to teach health education through life skills.

**Results:**

The variables explained 25% of the total variance in the ability to teach health education through life skills. When controlled in individual layers, the background variables and the enabling, reinforcing, and predisposing factors demonstrated explanatory powers of 6, 5, 7, and 7%, respectively, with respect to the ability to teach health education by utilizing life skills.

**Conclusions:**

Enhancement of the enabling, reinforcing, and predisposing factors can improve preschool educators’ ability to teach health education through life skills. The support provided by the governmental policies for related training can facilitate the effective implementation of health promotion programs in kindergartens. Preschool educators must also receive on-the-job training to facilitate the effective transaction of the health education curriculum. Health classes centered on life skills in kindergartens are vital and must be incorporated into the curricula.

## Background

As of 2019, there are 6348 kindergartens in Taiwan, including 2045 public kindergartens (32.2%) and 4303 private kindergartens (67.8%) The overall kindergarten enrollment rate among children aged 2 to 5 years is 67.3%, with a total of 565,000 students [1]. Many health problems reported in Taiwan’s Children’s Medical and Health Policy Suggestions for 2030 remain to be addressed, including vision, dental caries, obesity, insufficient levels of activity, and technology addiction [2]. This indicates the importance promotion of health in the kindergartens.

In 2020, the Ministry of Health and Welfare and Ministry of Education of Taiwan partnered with local governments to develop and implement the Promoting Health in Kindergartens program. The three main schemes of the campaign are kindergarten health policies, preschool health techniques and behaviors, and parent communication and community resources, which merge the four to address the major health concerns of vision care, accident and injury prevention, diet and nutrition, and physical fitness [3]. The ability of preschool educators to develop the life skills of preschoolers as a part of health education and the participation of parents and children are crucial for the successful implementation of this campaign. Article 3 of the Statute for Preschool Educators states that a preschool educator “refers to preschool staff, including principals, teachers, educare givers, and educare assistants” [4].

The early implementation of health education mainly relied on the dissemination of information and facts. The education method gradually shifted to focus on skill development with an emphasis on physical, social, emotional, and mental health, and on imparting health education during early childhood to facilitate the development of active and healthy behavior in young children [5]. Life skills training encourages individuals to make positive choices that can have subsequent health benefits and reduce dangerous and unhealthy behaviors; this training can be integrated with different topics in health classes [6]. Life skills refer to an individual’s ability to effectively manage the demands and challenges of various situations in their daily life [7]. In skills-based health education, life skills can be categorized as follows: (1) decision-making and problem-solving methods, (2) critical and creative thinking, (3) communication and interpersonal relationships, (4) self-awareness and empathy, and (5) stress and emotional management [8]. Many studies on teaching life skills and health to elementary school children or older children have been conducted in Taiwan, yet no study has focused on preschool-aged children.

The current study utilizes the perspective of preschool educators to investigate whether health classes based on life skills should be implemented at the preschool level, and explores the health education competence of the preschool educators.

Previous studies on preschool educators teaching in health-related fields have focused on the teaching abilities related to physical movement or motor skills [7, 9, 10], they have failed to discuss the influencing factors of teaching abilities in health domains. This study also analyzes the awareness of health learning indicators in preschool educators, specifically within the education curriculum framework on physical and health education [11]. The critical factors include preschool educators’ ability and cognitive capacity to guide health promotion and develop life skills in the children studying in kindergartens [5, 6, 8, 12–16] as well as health education knowledge of the educators [17, 18].

This study used the Predisposing, Reinforcing, and Enabling Constructs in Educational Diagnosis and Evaluation (PRECEDE) model as its theoretical framework. The model involves proposing an evaluation framework, collecting data, and developing a diagnostic tool to evaluate health education from an educational and environmental diagnostic perspective. The PRECEDE model can prioritize the results of interest and investigate active or passive effects [19] to guide the planning and development of interventional measures for healthy behaviors [20].

Based on the literature, the PRECEDE model [21] was used to investigate the factors that influence the preschool educators’ ability to teach life skills and health education. The three independent variables were: (1) predisposing factors that included: the preschool educators’ awareness of health learning indicators within the educational curriculum framework provided by the Ministry of Education [11], and the educators’ knowledge and cognition of both health education and its promotion in kindergartens [15–17] and life skills [5, 6, 8, 12–14]; (2) enabling factors that included: the evaluation of schools’ arrangement of health education, the extent to which kindergartens prioritize health modules and thematic teaching, the use of health education methods provided by social or official sources, and the convenient acquisition of resources (based on the techniques, materials, or equipment involved in the evaluation of the thematic health education); and (3) reinforcing factors comprising: the support of the kindergartens, colleagues, government policies, professional consultation services, parents, and the community [22–25]. The dependent variable in this study was the preschool educators’ ability to teach health education through life skills, and was measured through respondents’ self-assessment of their ability to strategically incorporate life skills into health education.

## Method

### Sample

This cross-sectional study’s participants were the preschool educators employed in the registered public or private kindergartens in Taipei City and New Taipei City. The participants and regions were selected based on the 2019 survey of kindergartens in Taiwan, which reported that public and private kindergartens in Taipei City and New Taipei City (hereafter referred to as the Greater Taipei Region) accounted for 28% (i.e., nearly one-third of all kindergartens in Taiwan). This region contained educators from 3875 and 12,982 public and private preschools respectively [1]. The selected participants included certified preschool educators who were currently employed as kindergarten principals, teachers, educare givers or assistants and had at least 6 months of related experience (excluding substitute, part-time, and intern teachers).

**Fig. 1:**
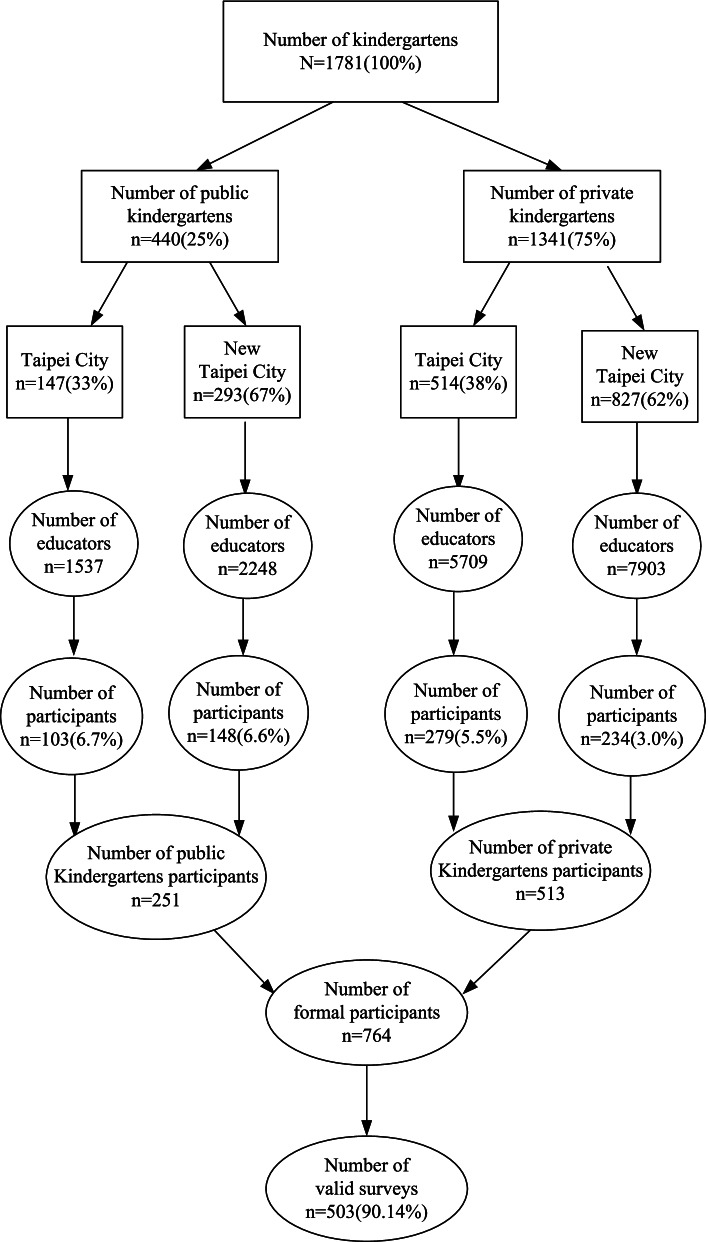
Formal samples stratified random sampling

The survey questionnaire used in the study has been depicted in Fig. 1. The sample was selected using stratified random sampling. The first level of sampling was based on the proportion of public and private kindergartens, which was 25 and 75%, respectively, while the second level was based on the administrative districts, with kindergartens drawn by lottery, and a 95% confidence interval and ± 5% margin of error. Therefore, the sampling involved 12 and 29 administrative districts in Taipei City (147 public kindergartens, 514 private kindergartens) and New Taipei City (293 public kindergartens, 827 private kindergartens), respectively. The number of preschool educators in the kindergartens varied because of the differences in the levels of the kindergartens. Using proportionate sampling, 103 and 148 questionnaires were sent to Taipei City’s public and private kindergartens respectively, while 279 and 148 questionnaires were sent to New Taipei City’s public and private kindergartens respectively.

The data used in this study were reviewed and approved by the Research Ethics Review Committee of the National Taiwan Normal University (Case number 201810HS025). This study was conducted from February to August 2019. The study respondents comprised 553 preschool educators from the Greater Taipei Region, with 50 participating in the pilot survey and 503 participating in the formal survey.

### Research tools

With the PRECEDE model serving as the theoretical framework [19–21, 26], a structured questionnaire was developed. The survey questions were validated and revised to generate the final survey questionnaire.

The structured questionnaire comprised five sections.

Table 1 depicts the first section that focused on the participants’ background (4 items) and presents information regarding the location of the kindergarten, registration in the kindergarten, level of education, and years of service.

**Table 1:** Structured questionnaire framework

Variables	factors	items	Scoring method	Overall Scoring	Reliability	Validity/ test analysis
Independent	Background	4	Category			
	Predisposing	16	5-point Likert scale	5–80	α: .964	X^2^: 18.51*** RMSEA < 0.001, CFI: 1.00 (SRMR) < 0.001
		12	1 or 0 points for correct and incorrect answers, respectively	0–12	KR20:.320	Difficulty:0.63
		15		0–15	KR20:.557	
	Enabling	5	multiple response items	0–37	KR20:.863	X^2^: 68.05*** RMSEA< 0.001, CFI: 1.00 SRMR < 0.001
	Reinforcing	5	multiple response items	0–39	KR20:.731	X^2^: 7.76** RMSEA< 0.001, CFI: 1.00 (SRMR) < 0.001
Dependent	Ability to teach	17	5-point Likert scale	5–85	α: .938	

** *p* < .01; *** *p* < .001

The second section focused on the predisposing factors (43 items) and included the following: (1) Preschool educators’ awareness of the health learning indicators within the education curriculum framework in reference to the preschool curricula [11]; (2) Preschool educators’ cognition of health promotion in kindergartens and life skills in reference to the six major constructs and strategic applications of health promotion in schools, as detailed by the WHO [16] and Hsiao [15]; and (3) Preschool educators’ evaluation of the knowledge pertaining to health education in reference to a prior questionnaire that surveyed teachers on health promotion in schools [17].

The third section of the questionnaire addressed the enabling factors (5 multiple-choice items, 37 obtained responses; each response was awarded 1 point). Subsection 1 explored the arrangements made by the school regarding provision of opportunities pertaining to health education. Subsection 2 procured information pertaining to the extent to which the kindergartens prioritized health modules or topics. Subsection 3 consisted of health education methods provided by social or official sources. Subsection 4 comprised health-related skills that can be developed and assessed. Subsection 5 addressed the acquisition of convenient materials or equipment resources.

The fourth section focused on the reinforcing factors (5 multiple response items, 39 obtained responses; each response was awarded 1 point). Subsection 1 procured information pertaining to the level of support offered by the kindergarten. Subsection 2 sought information on the level of support received from colleagues. Subsection 3 documented the perceived support from government policies. Subsection 4 focused on the professional consultation services and had five items on hiring experts as consultants. Subsection 5 sought information regarding the perceived level of support from parents and the community.

The fifth section addressed the educators’ ability to impart life skills and health education and comprised 17 items which were responded to on a 5-point Likert scale, developed based on the literature on life skills [5, 6, 8, 12–14]. A high score indicated that the respondents could positively assess the ability to strategically incorporate life skills in health education. The items investigated the ability of the preschool teachers with respect to the following life skills: self-awareness, decision-making, self-health management, critical thinking, and rejection.

Nine experts from the health and life skills education-related fields were invited to review the validity of the questionnaire. These preschool education experts, senior preschool educators, and pedagogues from the health education background were asked to assess the questions based on their suitability, clarity in the descriptions of items, and the applicability of the options using bi-sectioning; this enabled the calculation of the consistency as a percentage from the ratings of each item. In the question review analysis, the types of questions were selected based on their frequency distributions and percentages. Items designated as “appropriate” or “appropriate after revision” if they had a rating equal to or greater than 80% were retained [27, 28].

After the questionnaire was administered, an analysis of the relevant factors that affected the preschool educators’ ability to implement health education through life skills was conducted.

### Research steps

The pre-tests were validated and analyzed based on the assessment of the questionnaire by the experts. During the first phase, the questionnaire was administered as a pilot survey and revised based on the results. The second phase consisted of the final administration of the survey questionnaire followed by an analysis of the relevant factors that affected the preschool educators’ ability to implement health education through life skills. We conducted correlational analysis, t-tests, analysis of variance (ANOVA), and hierarchical regression. The research procedure has been described below.

#### Pilot survey

Preschool educators in the Greater Taipei Region were selected using intentional sampling. Only preschool educators were included in the pilot study sample. All 50 educators who were invited to participate provided valid samples (100% recovery rate).

A Factor analysis reviewed the reliability and validity of the items included in the pilot survey. The results indicated that communality was uniformly greater than 0.3, and the Cronbach’s α was 0.942 for the overall survey. Cronbach’s α for each construct was 0.907 for the predisposing factors, 0.920 for the enabling factors, 0.805 for the reinforcing factors, and 0.950 for the ability to teach health education through life skills.

#### Formal survey

After the questionnaire had been revised based on the pilot survey’s results, we used stratified random sampling to recruit the preschool educators from the Greater Taipei Region to participate in the survey. As in the case of the pilot survey, the research participants in the final survey were also preschool educators. After the educators in each kindergarten were contacted and consent was obtained, 764 survey questionnaires were dispatched and 558 responses were obtained (73.04% recovery rate).

After eliminating the invalid surveys, 503 questionnaires were analyzed; thus, the availability rate of the formal survey was 90.14%. Table 1 depicts the Cronbach’s α as 0.964 for the predisposing factors and 0.938 for the ability to teach health education through life skills. The Kuder-Richardson Formula 20 (KR20) was 0.320 for the “predisposing factors-cognition of health promotion in kindergartens and life skills,” 0.557 for the “predisposing factors-knowledge of health education,” 0.863 for the “enabling factors,” and 0.731 for the “reinforcing factors.” The test analysis results revealed the item difficulty value as 0.63 for the “predisposing factors.” The confirmatory factor analysis results were as follows for the “predisposing factors”: X^2^ = 18.51, *p* < 0.001, root mean square error of approximation (RMSEA) < 0.001, content validity index (CFI) = 1.00, and standardized root mean square residual (SRMR) < 0.001. For the “reinforcing factors” the results were as follows: X^2^ = 7.76, *p* < 0.01, RMSEA < 0.001, CFI = 1.00, and SRMR < 0.001; and for the “enabling factors”: X^2^ = 68.05, *p* < 0.001, RMSEA < 0.001, CFI = 1.00, and SRMR < 0.001. These results were consistent with the generally accepted rule of X^2^: *p* < 0.05, RMSEA < 0.08, CFI > 0.9, and SRMR < 0.08 [29], indicating that the predisposing, reinforcing, and enabling factors had good validity.

### Statistical methods

This data was analyzed using IBM SPSS for Windows 22.0, and Stata 14.0 was employed for conducting factory analysis, determining the internal consistency, and for carrying out reliability analysis based on Cronbach’s α coefficient and KR20 reliability. Descriptive statistics (distribution of frequency, percentages, mean values, and standard deviations), test analysis, and inferential statistics (t-test, ANOVA, Pearson’s correlation coefficient, and hierarchical regression) were also computed to verify the statistical significance of the results.

## Results

### Background information of the preschool educators

Table 2 shows that 259 (51.5%) and 224 (48.5%) respondents are employed at kindergartens in New Taipei City and Taipei City, respectively, with 305(60.6%) employed at private kindergartens and 198(39.4%) employed at public kindergartens. Most of the respondents (i.e., 431 [85.7%]) had a university-level education or above. With respect to years of service, 191 (38%) respondents had less than 10 years (38%).

**Table 2:** Respondents’ backgrounds information (*N* = 503)

Background variable	Group	N	Percentage (%)
Kindergarten location	New Taipei City	259	51.5
	Taipei City	244	48.5
Kindergarten registration	Private kindergarten	305	60.6
	Public kindergarten	198	39.4
Level of education	University or above	431	85.7
	Junior college or below	72	14.3
Years of service	Less than 10 years	191	38.0
	20 years +	159	31.6
	10 to 20 years	153	30.4

### Predisposing, enabling, reinforcing factors, and the ability to teach health education through life skills

Table 3 shows that the mean score of the 17-item of the questionnaire “Ability to teach health education through life skills” is 58.88 points, with a standard deviation (SD) of 9.92. The mean score among the individual items is 3.93 and the SD is 0.82. The item having the highest score was “I am able to teach preschoolers to use the life skills pertaining to management of self-health in order to maintain environmental hygiene (e.g., cleaning up the area after meals, and putting away toys)”,” with a mean score of 4.27 and SD of 0.67.

**Table 3:** Ability to teach health education through life skills (*N* = 503)

Item		Mean score	SD	
1. I can teach preschoolers to use the self-health management life skill of maintaining environmental hygiene (e.g., tidying up after meals, cleaning, and putting away toys).		4.27	0.67	
2. I can teach preschoolers to use the life skill of self-health management through correct personal hygiene practices (e.g., washing their hands and blowing their nose).		4.19	0.774	
3. I can teach preschoolers to use the life skill of self-health management to brush their teeth after meals at school.		4.06	0.847	
4. I can teach preschoolers to use the self-awareness life skill of observing the weather and temperature and appropriately responding by putting on more clothes or taking off clothes independently.		4.04	0.754	
5. I can teach preschoolers to use the self-awareness life skill of observing dangers and safety needs in their environment.		3.97	0.779	
6. I can teach preschoolers to use the self-awareness life skill of noticing when their eyes are sore, puffy, or aching, and respond by resting their eyes from staring at screens.		3.48	0.942	
7. I can use the decision-making life skill of teaching preschoolers to maintain healthy eating habits (e.g., chewing slowly, eating a set amount, and staying in their seat until they have finished eating).		4.13	0.801	
8. I can teach preschoolers to use the decision-making life skill of playing outside rather than remaining in the classroom during breaks.		3.67	0.973	
9. I can teach preschoolers to use the decision-making life skill of choosing a diet with less oil, salt, and sugar.		3.65	0.924	
10. I can use the critical thinking life skill of teaching preschoolers to recognize safe and fresh foods and to eat more vegetables and fruits that are rich in vitamins A, B, and C.		3.83	0.844	
11. I can use the critical thinking life skill of teaching preschoolers to differentiate between safe and unsafe actions and to maintain their distance from other people or objects when playing or moving around to avoid collisions.		3.95	0.733	
12. I can use the effective communication life skill of teaching preschoolers to use their words or bodies to express information to other people (e.g., vocalizing their needs or performing appropriate actions).		4.18	0.682	
13. I can use the interpersonal life skill to teach preschoolers to form friendships and interact politely with each other.		4.11	0.742	
14. I can use the rejection life skill to teach preschoolers not to interact with strangers and to protect themselves through proper daily hygiene practices (e.g., genital cleanliness, wearing of underwear).		4.06	0.82	
15. I can teach preschoolers to use the rejection life skill to protect their eyes (e.g., not staring at televisions, technology products, or video games with small screens).		3.29	1.02	
Overall mean	Overall standard deviation	Mean among items	Standard deviation among items	
58.88	9.92	3.93	0.82	

Table 4 shows the scores of the “predisposing, enabling, and reinforcing” constructs. The highest individual item score was recorded in the enabling construct of “Extent to which the kindergarten prioritizes health modules or topics” with a mean score among items of 7.02.

**Table 4:** Scores on predisposing, enabling, and reinforcing factors (*N* = 503)

Construct	Item type	Item	Total no. of items	Overall mean score	Overall SD	Mean among items	SD among items
Predisposing factors		Awareness of health learning indicators.	16	55.70	7.99	3.48	0.62
		Cognition of health promotion in kindergartens and life skills.	12	6.50	3.15	0.90	0.28
		Knowledge of health education	15	10.83	6.25	0.72	0.47
**Construct**	**Item type**	**Item**	**Total no. of items**	**Total times selected**	**Percentage of total times selected (%)** **Total sample size**	**Mean among items**	
Enabling factors	Multiple selection	Kindergartens’ arrangement of health education opportunities.	5	1572	315.0	3.15	
		Extent to which the kindergarten prioritizes health modules or topics.	12	3516	701.8	7.02	
		Use of health education methods provided for reference.	7	2025	405.8	4.06	
		Provision of skills that can be taught and assessed based on health themes.	5	1537	306.8	3.07	
		Acquisition of convenient materials or equipment resources	8	2624	527.1	5.27	
Reinforcing factors	Multiple selection	Support from parents and the community.	7	1522	305.6	3.06	
		Support from colleagues.	12	2502	501.4	5.01	
		Professional consultation services.	5	1020	206.5	2.07	
		Support from government policies.	5	1001	201.8	2.02	
		Support from the kindergarten.	10	1160	246.3	2.46	

### Correlations between the background, predisposing, enabling, and reinforcing factors and the variables pertaining to the ability to impart health education through life skills

Table 5 shows the correlation matrix of predisposing, enabling, and reinforcing factors and the ability to teach health education through life skills. The correlations: “Awareness of health learning indicators” (γ = − 0.32; *p* < 0.001), “Cognition of health promotion in kindergartens and life skills” (γ = 0.12, *p* < 0.01), “Knowledge of health education” (γ = 0.20; *p* < 0.001), “Support from the kindergartens” (γ = − 0.21; *p* < 0.001), “Support from government policies” (γ = 0.27; *p* < 0.001), “Extent the kindergarten prioritizes health modules or topics” (γ = 0.25; *p* < 0.001), “Use of health education methods provided for reference” (γ = 0.11; *p* < 0.05), “Provision of skills that can be taught and assessed based on health dimensions” (γ = 0.10; *p* < 0.05), and “Acquisition of convenient materials or equipment resources” (γ = 0.12; *p* < 0.05) showed statistically significant differences.

**Table 5:** Correlation matrix of predisposing, enabling, and reinforcing factors and the ability to teach health education through life skills (*N* = 503)

Variables	1	2	3	4	5	6	7	8	9	10	11	12	13	14	
1. Awareness of health learning indicators.	1														
2. Cognition of health promotion in kindergartens and life skills.	.03	1													
3. Knowledge of health education.	.20***	.19***	1												
4. Support from the kindergarten.	−.15**	.04	−.10*	1											
5. Support from government policies.	.15**	.06	.15**	˗.014	1										
6. Support from colleagues.	−.01	.03	.03	.23***	.18***	1									
7. Professional consultation services.	−.01	.05	.12**	.21***	.19***	.25***	1								
8. Support from parents and the community.	.04	.11*	.03	.34***	.26***	.41***	.39***	1							
9. Kindergartens’ arrangement of health education opportunities.	.12**	.01	.04	.07	.20***	.16***	.08	.16***	1						
10. Extent to which the kindergarten prioritizes health modules or topics.	.19***	.07	.12**	−.02	.50***	.14**	.09	.22***	.36***	1					
11. Use of provided health education methods for reference.	.19***	.03	.09*	.07	.42***	.16***	.14**	.26***	.44***	.60***	1				
12. Provision of skills that can be taught and assessed based on health themes.	.14**	.01	.18***	.08	.37***	.20***	.21***	.25***	.32***	.40***	.55***	1			
13. Acquisition of convenient materials or equipment resources.	.24***	.05	.12**	.09*	.38***	.20***	.16***	.27***	.42***	.54***	.63***	.57***	1		
14. Ability to teach health education through life skills.	.32***	.12**	.20***	˗.21***	.27***	.06	.02	.06	.05	.25***	.11*	.10*	.12*	1	

* *p* < .05; ** *p* < .01; *** *p* < .0

The t-test and ANOVA results indicate “Kindergarten registration” (*t* = 2.07; *p* < 0.05) and “Level of education” (F = 9.08; *p* < 0.001) are significantly different. Variables with significant differences were converted into dummy variables and entered into the hierarchical regression model.

Following the analysis results, the PRECEDE model was applied to enhance the continuity of healthy behaviors. The enabling factors acted as strong indicators which implied that they could be used as predisposing factors in the next behavioral phase. When a behavior is rewarded, reinforcing factors in this phase can also become predisposing factors in the next phase [29]. Therefore, in the analysis model, following the control for the related factors, we entered the background variables and the enabling, reinforcing, and predisposing factors into the four regression analysis models.

### Predictive models for “ability to teach health education through life skills” based on the background, predisposing, enabling, and reinforcing variables

This study further explored the effects of the independent variables (i.e., background [2 items], the enabling [4 items], reinforcing [2 items], and predisposing factors [3 items]), on one dependent variable “Ability to teach health education through life skills.”

Table 6 presents the results of the hierarchical regression analysis. According to Model 1, “Background factors” demonstrate significant explanatory power for “Ability to teach health education through life skills” (F = 16.02; *p* < 0.001). This result shows that the respondents perceive the preschool educators who have university-level education and work in a private kindergarten as being able to effectively teach life skills in health education.

**Table 6:** Hierarchical regression analysis of predisposing, enabling, and reinforcing factors and the ability to teach health education through life skills (*N* = 503)

Variables	Model 1		Model 2		Model 3		Model 4		
	Background factors		Background and enabling factors		Background, enabling, and reinforcing factors		Background, enabling, reinforcing, and predisposing factors		Collinearity diagnostic
	β	t	β	t	β	t	β	t	VIF
**Background variables**									
Kindergarten registration.	2.73	3.04*	2.94	3.22**	2.99	3.46**	3.20	3.86***	1.09
Level of education.	6.62	5.29***	5.45	4.34***	4.78	3.93***	3.61	3.04**	1.15
**Enabling factors.**									
Acquisition of convenient materials or equipment resources.			−.13	−.36	−.04	−.115	−.32	−.96	2.04
Provision of skills that can be taught and assessed based on health themes.			.40	.72	.21	.39	.24	.46	1.73
Extent to which the kindergarten prioritizes health modules or topics.			.94	4.53***	.60	2.83*	.55	2.71*	1.90
Use of provided health education methods for reference.			−.43	−.92	−.51	−1.12	−.57	˗1.31	2.16
**Reinforcing factors**									
Support from government policies.					.70	4.04***	.63	3.79***	1.43
Support from the kindergarten.					1.25	4.58***	.98	3.61***	1.07
**Predisposing factors**									
Aware of health learning indicators.							.30	5.80***	1.14
Cognition of health promotion in kindergartens and life skills.							.76	2.18*	1.05
Knowledge of health education.							.31	1.76*	1.15
R^2^	0.06		0.11		0.17		0.25		
Adj R^2^	0.06		0.10		0.16		0.23		
F	16.02***		10.16***		13.01***		14.60***		
△R^2^	0.06		0.05		0.07		0.07		
△F	16.02***		6.85***		19.31***		15.74***		

CN: 21.5

* *p* < .05; ** *p* < .01; *** *p* < .001

Model 2 incorporates “background factors” and “enabling factors.” The ability to explain the dependent variable increases to 11% (F = 10.16; *p* < 0.001). These findings indicate that the preschool educators perceive their kindergartens as being suitable for prioritizing the teaching of health modules or topics and that preschool educators are able to effectively teach health education using life skills.

Model 3 incorporates the “background factors” “enabling factors” and “reinforcing factors,” increase the explanatory power of the dependent variable to 17% (F = 13.01; *p* < 0.001), indicating that the preschool educators perceive support from government policies and the kindergarten as factors that assist preschool educators to effectively teach health education through life skills.

The three predisposing factors of Model 4, increase the explanatory power of the dependent variable to 25% (F = 14.60, *p* < 0.001). This indicates that the respondents perceive preschool educators who are aware of health learning indicators, cognizant of health promotion in kindergartens and life skills, and knowledgeable about health education as likely to be able to effectively teach life skills in health education.

A comparison of these four models revealed that Model 4 had the greatest explanatory power. We then performed collinear diagnostics to avoid collinearity in the regression equation violating the basic assumptions. The collinearity diagnostics revealed a variance inflation factor (VIF) smaller than 10 [30] and condition number (CN) smaller than 30 [31]; thus, the regression equation was determined not to demonstrate collinearity (Table 6).

## Discussion

### Health education by preschool educators based on the PRECEDE model

The results of the study revealed that the scores were high for the “Ability to teach health education through life skills” variable, indicating that the preschool educators were able to impart health education through life skills. Therefore, preschool educators are unlikely to oppose initiatives for health promotion in kindergartens that aim to incorporate health education strategies. This is consistent with the research results of Hanley et al. [32] and Fahmie and Luczynski [33].

The responses for the “Predisposing” construct indicated that although the preschool educators had medium to high levels of awareness of the learning indicators in the health curricula, and whether they could design learning activities in line with health topics that would allow preschoolers to gain correct health skills and behaviors requires further empirical study. The item with the highest scores within the scale measuring the cognition of health promotion in kindergartens and life skills was “Kindergarten health courses must be designed to incorporate the six major domains of the government’s educare curriculum framework (e.g., physical movement and health, cognition, language, society, emotions, and aesthetics) and administered in an integrated approach.” This demonstrates that the preschool educators universally reported the importance of designing health education classes on life skills that would incorporate these six educational domains. Early childhood education courses are designed along many axes, and each part covers multiple domains. Rather than being classified based on a particular subject or domain, they focus on a specific topic around which educators can conduct activities that offer different learning opportunities and guide their students to think about related problems [34].

Notably, for every item in the questionnaire on health knowledge of education, at least one preschool educator answered incorrectly or did not know the answer. Thus, updating and expanding preschool educators’ health knowledge is vital and this finding resonates with the existing research.

The results for the “Enabling” construct revealed that the items that had the highest frequency of responses pertained to the resources provided by society or official sources, such as teaching resources, administration of tools of assessments, and application of teaching methods. This was highly consistent with the indicators of professional competence among kindergarten teachers that described the need for flexible and engaging teaching methods to increase preschoolers’ interest and willingness to learn, as well as the use of diverse evaluation methods to assess learning outcomes [35]. Regarding the provisions of opportunities pertaining to health education in the kindergartens, most responses indicated that health education was most frequently imparted through “specific campaigns.” However, certain “Specific health modules or topics” (such as vision health, oral health, and sex education), which have not been specified in the Act but need to be emphasized, were associated with fewer educational campaigns. Wu argued that while creating health promotion programs for kindergarteners, health must be treated as a holistic concept, and that related initiatives should not only broaden the preschoolers’, educators’, and parents’ knowledge of health-related concepts but also provide an in-depth understanding, thereby increasing their awareness and leading to effective modification of their health-related behavior [35].

For the “Enabling” construct, the items which received the highest number of responses pertained to the support from parents and the community. This implied that preschool educators believe that raising parental and community awareness of health campaigns can facilitate the achievement of the health educators’ objectives. The item that received the second-highest number of responses concerned support from colleagues, which is crucial in kindergartens’ highly collaborative teaching programs. Support from professional consultation services, government policies, and kindergarten administrators failed to facilitate the accomplishment of kindergartens’ health education objectives and in improving the efficacy of health promotion activities. These results are similar to those of Niu et al. [22].

The results of this study indicate the willingness of preschool educators to teach health education and recognize its importance. However, the preschool educators’ health-related knowledge should be updated and expanded if necessary to enhance educators’ competence in teaching this subject. Effective training materials provided by the governmental agencies that emphasize a holistic concept of health rather than specific initiatives can broaden the health-related knowledge of educators, parents, and preschoolers. Additionally, support from both colleagues and parents is essential for successful promotion.

### Improvement of preschool educators’ ability to teach health education through life skills within the PRECEDE framework

We analyzed whether the intrinsic factors, such as initial motivation, affected the preschool educators’ teaching of health education through life skills taking the predisposing factors into account. Then, we examined whether the external factors, such as individual available resources and teaching techniques, affected the preschool educators’ teaching of health education through life skills. Finally, we considered the reinforcing factors to review the social factors, such as the emotional, mental, and social support provided by family, colleagues, teachers, and health providers, to determine how they affected the preschool educators’ teaching of health education through life skills. Since the PRECEDE model can further ensure continuity of a behavior, the enabling factors can serve as indicators and may play the role of predisposing factors in the next behavioral stage. When a behavior is rewarded, the reinforcing factors in the current stage can act as predisposing factors in the next stage [29].

In this study, “Ability to teach health education through life skills” demonstrated significant correlations with the predisposing factors of “Awareness of health learning indicators,” “Cognition of health promotion in kindergartens and life skills,” and “Knowledge of health education.” Owing to the lack of literature on health education in kindergartens, limited evidence is available to compare the findings. The findings of the study with respect to the positive effect of the predisposing, enabling, and reinforcing factors in improving preschool educators’ ability to teach health education through life skills are similar to the findings of Niu et al. [22].

During further investigation of the most influential factors, it was found that the predisposing factors can motivate preschool educators to enhance their ability to teach health education through life skills. Therefore, improving the predisposing factors could effectively strengthen the preschool educators’ ability to teach health education through life skills. In addition, by improving the reinforcing factors and the enabling factors preschool educators’ ability to teach health education through life skills can be enhanced. With respect to the background variables, the respondents perceived the preschool educators who had earned university degrees and positions in private kindergartens as being able to teach health education through life skills. This result may be attributable to the greater proportion of respondents who were employed in private kindergartens, accounting for most of the kindergartens in Taiwan. Another possibility is that private kindergartens provide more flexibility in teaching, which is likely to influence the teaching of health education through life skills. The partiality towards having a university-level education or higher indicates that a high level of education is perceived as being crucial for the promotion of health education through the teaching of life skills.

## Conclusions

There were significant correlations among the preschool educators’ ability to teach health education and the predisposing, reinforcing, and enabling constructs in the PRECEDE model.

The explanatory power of the regression model of preschool educators’ ability to impart health education using the predisposing, reinforcing, and enabling constructs increased (R2 = 25%).

This study was novel as it included preschool educators as a study sample. Enhancement of the enabling, reinforcing, and predisposing factors can improve preschool educators’ ability to impart health education through life skills. Support in the form of government policies that aim at providing training to the educators can facilitate the effective implementation of health promotion programs in kindergartens. On-the-job training facilities should also be provided to the preschool educators to facilitate effective transmission of the knowledge pertaining to health education. Health classes revolving around life skills in kindergartens are vital and must be incorporated into the curricula.

### Suggestions

Based on the results of the study, the following suggestions are being offered:

1. Government agencies

The results of the study indicate that support from governmental policies influences preschool educators’ “Ability to teach health education through life skills.” Therefore, government agencies can utilize this study’s findings while formulating policies that promote health in kindergartens. The 2020 Promoting Health in Kindergartens program in Taiwan offers training to preschool educators to improve their ability to impart health education by utilizing their life skills.

2. Future research

The results of the current study offer avenues for future research. Although it was found that the preschool educators had medium to high levels of awareness of the learning indicators in the health curricula, further studies are required to determine whether they are capable of designing learning activities in line with the health-related topics that enable preschoolers to develop the necessary skills and behavior to lead a healthy life.

### Limitations

This study also had some limitations that are likely to affect the generalizability of the results. Due to the restrictions pertaining to labor, cost, time, and willingness of the respondents, this study was geographically restricted to the Greater Taipei Region. Further, the sample characteristics and demographic variables of this region may differ considerably from those of other regions. These factors are likely to affect the easy conceptualization and generalizability of the results to the other regions.

Additionally, the study design utilized a survey method of data collection which is limited by selection and response biases. In future surveys, utilization of a “not applicable” field and asking the non-respondents to participate in a follow-up survey are strategies to mitigate selection bias. To avoid response bias, nine experts from related fields were invited to review the validity of the study questionnaire.

## Data Availability

The datasets generated and/or analyzed during the current study are not publicly available because of the regulations of the Institutional Review Board of the National Taiwan Normal University’s Research Ethics Review Committee. However, the datasets are available from the corresponding author upon reasonable request.

## Abbreviations

ANOVA: Analysis of variance
CFI: Comparative fit index
CN: Condition number
PRECEDE: Predisposing, Reinforcing, and Enabling Constructs in Educational Diagnosis and Evaluation
PPM: Parts per million
RMSEA: Root mean square error of approximation
SD: Standard deviation
SRMR: Standardized root mean square residual
VIF: Variance inflation factor
IBM SPSS: International Business Machines Corporation Statistical Product and Service Solutions
STATA: Software for Statistics and Data Science
WHO: World Health Organization
